# First Report of *Curtobacterium flaccumfaciens* in Bulgaria

**DOI:** 10.3390/pathogens13060483

**Published:** 2024-06-06

**Authors:** Yoana Kizheva, Maria Pandova, Melani Dimitrova, Yoana Gladicheva, Maria Garkova, Desislava Pirnareva, Deyan Donchev, Penka Moncheva, Petya Hristova

**Affiliations:** 1 Department of General and Industrial Microbiology, Faculty of Biology, Sofia University, 1164 Sofia, Bulgaria; maria.pandova@abv.bg (M.P.); melanievtimova72@gmail.com (M.D.); y.gladicheva@abv.bg (Y.G.); mariabt16@yahoo.com (M.G.); desi_pir@abv.bg (D.P.); montcheva@abv.bg (P.M.); pkabad@biofac.uni-sofia.bg (P.H.); 2 National Reference Laboratory for Control and Monitoring of Antimicrobial Resistance, Department of Microbiology, National Center of Infectious and Parasitic Diseases, 26 Yanko Sakazov Blvd., 1504 Sofia, Bulgaria; deyandonchev@ncipd.org

**Keywords:** *Curtobacterium flaccumfaciens*, alternate host plants, antimicrobial substances, phytopathogenic coryneform bacteria

## Abstract

This study aims at the identification and characterization of five actinobacterial strains with presumed belonging to the species *Curtobacterium flaccumfaciens* isolated from tomato and pepper plants, and establishing the potential role of both plants as natural reservoirs of this phytopathogen. Species identification was performed via MALDI-ToF MS, 16S rDNA sequencing and PCR. The strains were Gram-positive with a coryneform cell shape having yellow/orange-pigmented colonies; positive for catalase and esculin, and starch and casein hydrolysis; oxidase-, urease-, indole- and nitrate-reduction-negative and were strictly aerobic. All isolates produced antimicrobial substances against various phytopathogenic bacteria. Tomato and pepper plants were artificially infected with newly isolated strains in order to establish their role as natural reservoirs of the bacteria. Morphological alterations were observed only in the tomato plants, with defoliation of the first two to four leaves at the 28th day. Then, viable coryneform bacterial isolates (n = 73) were successfully re-isolated only from the stems of the infected plants. The similarity between the re-isolates and the respective initial isolates was confirmed phenotypically and genotypically by RAPD-PCR, confirming that solanaceous vegetables can act as reservoirs of *C. flaccumfaciens*. This is the first report of *C. flaccumfaciens* in Bulgaria.

## 1. Introduction

Members of the family *Microbacteriaceae* (order *Actinomycetales*, class *Actinobacteria*) are known to inhabit diverse ecological niches (aqueous and terrestrial), having an epiphytic, endophytic or pathogenic lifecycle [1]. They are aerobes with a Gram-positive pleomorphic cell shape (coryneform) and a high G+C content. The species comprise characteristics, like a specific B-type cell wall structure and unsaturated respiratory menaquinones, which separates them from the other actinobacteria [2]. According to the List of Prokaryotic names with Standing in Nomenclature (LPSN), the family consists of more than 60 genera [3] (accessed in May 2024).

The genus *Curtobacterium* is a member of the family *Microbacteriaceae* and was first described by Yamada and Komagata in 1972. It includes pathogenic and non-pathogenic species [4]. According to International Committee on Systematics of Prokaryotes (ICSP) and the International Code of Nomenclature of Prokaryotes (ICNP), the current composition of the genus comprises of 18 species with validly and not validly published names [3,4,5,6,7,8,9,10,11,12,13].

Among these species, two are known as plant pathogens, *Curtobacterium flaccumfaciens* (*Cf*) and the recently described *Curtobacterium allii,* causing onion bulb rot [13,14]. The latter species has been recently proposed for reclassification as a pathovar of *C. flaccumfaciens* [14]. The species *C. flaccumfaciens* includes pathogenic and non-pathogenic strains [15] as well as strains associated with human diseases [16,17]. On the other hand, data regarding the role of *C. flaccumfaciens* as a plant-beneficial endophyte, reducing disease symptoms, has also been reported [18,19]. Several pathovars of the species are known to be pathogenic and to cause bacterioses on various plants, including *C. flaccumfaciens* pv. *ilicis* (bacterial blight of American holly), *C. flaccumfaciens* pv. *betae* (silvering disease of red beet), *C. flaccumfaciens* pv. *oortii* (bacterial wilt and spot of tulip), *C. flaccumfaciens* pv. *poinsettiae* (bacterial canker of poinsettia) and *C. flaccumfaciens* pv. *flaccumfaciens* (*Cff*) (bacterial wilt on dry bean) [15]. Due to its seed-borne nature, the latter pathovar of the species is considered a pathogen of high risk and is included in the A2 list of quarantine pests by the EPPO [20]. Additionally, the pest is under strict control as a dangerous microorganism and is prohibited from entering and spreading throughout the protected zones of Greece, Portugal and Spain if it is found on *Phaseolus vulgaris* (common bean) and *Dolichos* seeds [21].

Apart from its common habitats, representatives of the species *C. flaccumfaciens* have been isolated from diverse plant species that are not among its known susceptible hosts. The role of such alternative and alternate host plants as natural reservoirs of this coryneform bacterium is considered of great importance and has recently been the subject of serious investigation. Among them, tomato, bell pepper, eggplant, sunflower, wheat, etc., could be mentioned [22,23,24]. In this regard, clarifying the ability of the pathogenic bacteria to persist and survive in various uncommon habitats sheds light on the probability that they will exert their pathogenic potential and expand their host range. Supporting this hypothesis is a study reporting that two strains of *C. flaccumfaciens* have caused chlorosis and leaf death on maize under greenhouse experiments [22]. Since maize is not one of *C. flaccumfaciens*’ known susceptible hosts, this shift in the bacterial pathogenicity is quite concerning and has prompted the need for additional research in this area.

Over the past few decades, knowledge about the composition of *C. flaccumfaciens* has accumulated, demonstrating that it is a complex species. In most of the cases, the identification at the species level has been conducted via polyphase approaches based on the comparison of the sequences of 16S rDNA [25,26,27,28,29,30], MALDI-ToF MS [17,28,29] and PCR methods [22,23,30]. The most applied PCR protocols are usually based on amplification with pathovar-specific primers specifically targeting *C. flaccumfaciens* pv. *flaccumfaciens* [31,32]. Various molecular techniques have been conducted over the years aimed at establishing the genetic structure inside the species. As a result, great genetic diversity has been established based on DNA fingerprint methods, such as AFLP, rep-PCR and PFGE, and multilocus sequence analyses (MLSAs) [15,22,33,34,35]. Recently, a significant reclassification within the species *C. flaccumfaciens* has been proposed based on the comparison of the whole-genome sequences of numerous *Curtobacterium* strains and the evaluation of the average nucleotide identity (ANI) and digital DNA–DNA hybridization [14]. The orange-pigmented strains of *C. flaccumfaciens* pv. *flaccumfaciens* have been reclassified as *Curtobacterium aurantiacum* sp. nov., the orange- and pink-pigmented strains of *C. flaccumfaciens* pv. *poinsettiae* were reclassified as *C. poinsettiae* sp. nov., *C. allii* [13] was reclassified as *C. flaccumfaciens* pv. *allii* pv. nov. and *Curtobacterium flaccumfaciens* pv. *basellae* [36] was reclassified as *Curtobacterium citreum* pv. *basellae. C. citreum* and *Curtobacterium albidum* were considered the same species [14].

Currently, *C. flaccumfaciens* and *Cff* in particular are considered absent in Bulgaria according to The Bulgarian National Plant Protection Organization (Service) (NPPO), although a few records concerning the pathogen have been published almost 40–50 years ago by Radkov and Nedelchev in 1975 and Boyadzhiev and Kakumkova in 1985, according to the EPPO Global Database [37]. Since then, the species has not been reported in Bulgaria, and this study is the first report for the last almost five decades. Thus, the main aim of this study is to announce, for the first time in Bulgaria, the isolation and species identification and characterization of newly isolated bacterial strains with presumed belonging to the species *C. flaccumfaciens*. The species was found accidentally on tomato and pepper plants with symptoms of bacterioses, and our purpose was to establish the potential role of *Solanum lycopersicum* (cv. Ideal) and *Capsicum annuum* (cv. Zlaten medal) as natural reservoirs of this bacterium.

## 2. Materials and Methods

### 2.1. Bacterial Strains Used in This Study

For the aims of this study, several species of bacteria were used as test microorganisms in the experiment for the production of antimicrobial substances (AMSs): phytopathogenic bacteria *Xanthomonas euvesicatoria* strains 40f (tomato flower isolate), 269p (pepper isolate) and 105t (tomato isolate); *Xanthomonas vesicatoria* strain 124t (tomato isolate) and *Xanthomonas gardneri* strain 62t (tomato isolate), which have been previously characterized and their pathogenicity to sensitive plants (tomato and pepper) has been evaluated [38,39,40]; *Clavibacter michiganensis* subsp. *michiganensis* CFBP 2492; and plant-associated bacterium *Pseudomonas putida* ATCC 12633. The type strain *C. flaccumfaciens* pv. *oortii (Cfo)* CFBP 1384 was also included in the analyses as the control bacterial strain. 

### 2.2. Plant Material and Isolation of Coryneform Bacterial Strains

**Figure 1:**
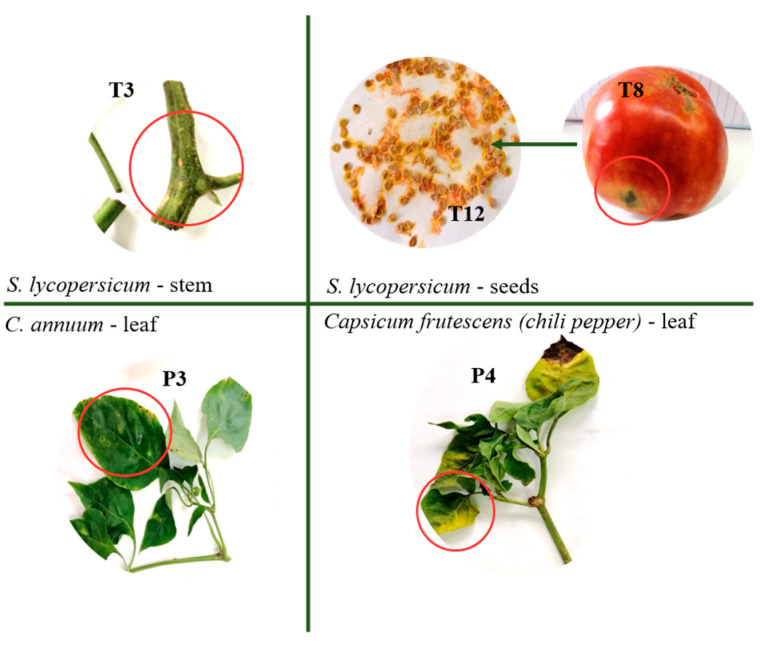
Plant samples used for the isolation of the bacteria.

Plant samples (*S. lycopersicum* L, *C. annuum* L and *Capsicum frutescens*) with symptoms of diseases were randomly collected from a crop field and used for phytopathogenic bacteria isolation [41]. The different parts of the diseased plants (Figure 1) were separated, rinsed with sterile dH_2_O and processed with iodine alcoholic solution to remove the epiphytic microflora. 

The zones with disease symptoms but surrounded by healthy tissue were cut off with sterile scissors, homogenized in sterile saline (NaCl, 0.9%), incubated for 30 min at room temperature and periodically vortexed every 5 min. The resulting suspensions were 10-fold diluted in sterile saline and 100 µL of each dilution was surface plated on yeast dextrose calcium (YDC) agar (HiMedia, Mumbai, India). The Petri dishes were cultivated aerobically at 28 °C for 48–72 h. The initial selection was performed based on the colony morphology formed on the YDC agar after cultivation—yellow or orange pigmentation, shiny and smooth. Pure bacterial cultures were obtained after the double re-cultivation of selected single colonies on potato sucrose agar (PSA) (potato extract, 200 g/L; sucrose, 20 g/L; agar, 15 g/L; dH_2_O, 1 L; pH = 7.0). The obtained bacterial strains were stored at 4 °C for further analyses.

### 2.3. Phenotypic Characterization of the Bacterial Isolates

Several key biochemical characteristics, recommended by the EPPO, were studied for all newly isolated strains [20]: aerobic/anaerobic growth, hydrolysis of esculin, starch and casein, production of urease, catalase and cytochrome c oxidase, production of indole from tryptophan and nitrate reduction as well as Gram staining. Log-phase cultures (obtained after overnight cultivation at 28 °C on PSA) were used for the determination of phenotypic characteristics. Bacterial suspensions, 10^8^ CFU/mL (MacFarland units, 1.3), were prepared in normal saline and used for the analyses. The ability for esculin hydrolysis was determined on bile esculin agar (Bioprepare Microbiology, Markopoulo, Greece). The ability for starch hydrolysis was evaluated on starch agar (beef extract, 3 g/L; peptone, 5 g/L; soluble starch, 10 g/L; agar, 15 g/L, dH_2_O, 1 L; pH of 7.5) and casein hydrolysis was evaluated on skim milk agar (beef extract, 3 g/L; peptone, 5 g/L; skim milk, 15 g/L; agar, 15 g/L, dH_2_O, 1 L; pH of 7.5). The motility, indole and urease medium (MIU medium) (LQ092, HiMedia, Mumbai, India) was used for the determination of urease and indole production. The catalase production was established by the addition of 3% H_2_O_2_ to the culture. For the determination of the production of cytochrome c oxidase, Bactident Oxidase test strips (Merck KGaA, Darmstadt, Germany) were used. The ability of the tested strains to reduce nitrates were evaluated on semi-solid nitrate media (peptone, 5 g/L; yeast extract, 4 g/L; agar 3 g/L; KNO_3_, 1 g/L; pH = 7.0). The inoculated media were cultivated at 28 °C for 48–72 h. The presence of either nitrates or nitrites after the cultivation period was tested colorimetrically using nitrate test strips (10–500 mg/L (NO_3_^−^) (Merck KGaA, Darmstadt, Germany).

### 2.4. Isolation of Chromosomal and Plasmid DNA from the Strains

All strains were aerobically cultivated overnight in 50 mL of Luria Bertani (LB) broth at 28 °C in a rotary shaker (RSLAB-7PRO, Auxilab, Polígono Morea Norte, Beriáin (Navarra), ESPAÑA) to obtain the log-phase cultures. For the isolation of chromosomal DNA (cDNA) from the strains, aliquots of one milliliter of the resulted bacterial cultures were centrifuged at 8000 × *g* for 10 min to collect the bacterial pellet. The cDNAs were extracted using commercial DNA isolation kits, the GenEluteTM Bacterial Genomic DNA Kits (Merck KGaA, Darmstadt, Germany). For the isolation of the plasmid DNAs from the strains, 5 mL of the resulted bacterial cultures were used and the bacterial pellets were collected as described above. The plasmids were extracted with the EZNA Plasmid DNA Mini Kit I (Omega Bio-Tek, Inc., Norcross, GA, USA) according to the recommendations of the manufacturer. The quality and quantity of the obtained DNAs were checked electrophoretically on 1% agarose gel and on the spectrophotometer/fluorometer DeNovix DS-11 FX+ (DeNovix Inc., Wilmington, DE, USA), respectively. For the complete lysis of the cells, 2 μL of 1000 units/mg mutanolysin (Merck KGaA, Darmstadt, Germany) was added at the enzyme lysis step. The isolated DNAs were stored at −20 °C and used in the further analyses.

### 2.5. Identification of the Isolated Bacteria

A polyphase approach was used for the identification of the newly isolated strains, with MALDI-ToF (Matrix-Assisted Laser Desorption/Ionization Time-of-Flight) mass spectrometry (MS) [41], 16S rDNA sequencing and PCR with primers CffFor2/CffRev4, specific for the detection of *Cff* [31]. The 16S rDNA genes of all isolates were amplified using the primer pair 27F/1492R [42]. PCR reactions were carried out in a total volume of 50 µL, containing 22 µL of ultra-pure H_2_O, 25 µL of PCR mixture 2 × iProof HF MasterMix (BioRad, Laboratories Inc., Sofia, Bulgaria), 1 µL of each primer (10 pmol/µL) and 1 µL of cDNA. The reaction conditions were as follows: initial denaturation at 94 °C for 7 min, followed by 30 cycles of 94 °C for 45 s, 57 °C for 1 min, 72 °C for 1 min and final elongation steps at 72 °C for 10 min. The PCR products were visualized by 1.5% agarose gel electrophoresis. Purified PCR products were sequenced in Macrogen Europe, Meibergdreef 57 1105 BA, Amsterdam, The Netherlands. The obtained sequences were subjected to comparative analyses using BLASTN on NCBI (accessed in April 2024) and 16S-based ID on EzBioCloud databases [43] (accessed in April 2024). The genome assemblies of a set of type and non-type strain members of the family *Microbacteriaceae* were downloaded from NCBI and their 16S rDNA genes were extracted. They were aligned together with the 16S rDNA sequences obtained from our strains using a Clustal Omega 1.2.2 using the mBed fast algorithm. A phylogenetic tree was built with FastTree v 2.1.11 using the Generalized Time-reversible genetic distance model. The resulting tree was annotated with iToL v 6.9 [44].

The pathovar-specific PCR (primers CffFor2/CffRev4) was performed according to Tegli et al. [31], and chromosomal and plasmid samples were used as DNA templates in separate reactions. 

All PCR reactions were carried out in MJ Research PTC-200, Peltier Thermal Cycler (Hampton, NH, USA).

### 2.6. Evaluation of the Antimicrobial Activity of the Isolates

The ability of our *C. flaccumfaciens* isolates to produce AMSs was carried out according to procedure described by Osdaghi et al. [22] with slight modifications. All strains isolated in this study along with the type strain *C. flaccumfaciens* pv. *oortii* CFBP 1384 were tested for extracellular production of AMSs against various phytopathogenic and plant-associated test bacteria (see Section 2.1). The potential producers were spot-inoculated on the surface of 20 mL of yeast peptone glucose agar (YPGA) medium (yeast extract, 7 g/L; peptone, 7 g/L; glucose, 7 g/L; agar 18 g/L; dH_2_O, 1 L) and incubated at 28 °C for 48 h. The test bacteria were cultivated on PSA until the obtainment of the log-phase culture, after which fresh bacterial suspensions with a concentration of 10^8^ CFU/mL (MacFarland units, 1.3) were prepared in normal saline. An amount of 200 µL of each suspension, separately, were mixed with 3 mL of soft YPGA (agar content, 4.5 g/L) medium and poured onto the surface of the Petri dishes with the previously cultivated AMS producers (our strains). The Petri dishes were cultivated for 48 h at 28 °C and the appearance of clear zones around the spots were considered as a positive result for AMS production from the respective strain. For the purposes of this study, the sensitivity of the test bacteria to the produced AMSs, determined on the basis of the diameter of the inhibitory zones, was described with the following 4 groups: ≥41 mm, strong sensitivity (SS); 21–40 mm, intermediate sensitivity (IS); 7–20 mm, weak sensitivity (WS); 0–6 mm, resistance (R). The experiments were performed in triplicate.

### 2.7. Artificial Infection of Tomato and Pepper Plants

Seeds of *S. lycopersicum* L. cv. Ideal and *C. annuum* L. cv. Zlaten medal were surface-disinfected with a 0,03% solution of KMnO_4_ and sown in a sterilized (130 °C for 30 min) commercial soil mixture (Agricult, St. Ioannou Theologou 115, Acharnai, Greece). The seedlings were grown in climatic chamber under controlled conditions (a 26 ± 2 °C temperature, a 16/8 h day/night photoperiod, a light intensity of 70–90 µmol/m^2^/s and 60–70% relative air humidity) until reaching the 3–4th leaf stage. The newly isolated bacterial isolates (3t_2_, 12t_1_, 3p_3_, 4p_1_ and 4p_2_) were grown in PSA at 28 °C overnight to obtain the log-phase cultures. The bacterial suspensions were prepared in sterile dH_2_O and adjusted to 10^8^ CFU/mL (MacFarland units, 1.3). The artificial infections of the plants were performed according to the procedure described by Osdaghi et al. [22]. Each plant was individually infected: tomato plants with strains 3t_2_ and 12t_1_ and the pepper plants with strains 3p_3_, 4p_1_ and 4p_2_. Sterile dissecting needles were dipped into the bacterial suspensions and, after that, the plants were pierced through the first internode. The control plants were treated the same way but, instead of bacterial suspensions, sterile dH_2_O water was used. The infected test plants were cultivated for a period of 28 days under the controlled climatic chamber conditions described above and observed for the appearance of any morphological changes every 7 days. The experiments were conducted in triplicate with three plants per each individual infection. 

### 2.8. Re-Isolation of C. flaccumfaciens Strains from the Artificially Infected Plants

After the examined period (28 days), the plants were analyzed for the presence of inoculated *C. flaccumfaciens* strains in the aboveground plants’ parts (leaves, stems and petioles). Each part of the particular plant was finely chopped with a scalpel and homogenized in sterile saline. The resulting homogenates were incubated at room temperature for 30 min with strong vortexing at every 5 min. After that, the suspensions were 10-fold diluted in normal saline and aliquots of 100 µL of each dilution were surface-inoculated on YDC agar. The Petri dishes were incubated at 28 °C for 24–72 h until visible bacterial growth was observed. All samples were carefully checked and only single colonies, corresponding to the initial bacterial isolates, were isolated on PSA. Pure cultures were obtained after two consecutive re-cultivations on PSA. The isolates were stored at 4 °C and used for further analyses.

### 2.9. Determination of the Similarity between the Initial Isolates and the Re-Isolates

Micromorphological, biochemical and molecular characterization was performed to determine the similarity between the initial bacterial isolates used for the artificial infection of the plants and the respective re-isolates. All re-isolated bacteria were Gram-stained and microscopically observed. Aerobic/anaerobic growth as well as catalase and oxidase activity were also determined. The RAPD-PCR approach was optimized for the purposes of this study in order to determine the unique molecular pattern of each initial strain. The similarity between the initial strains and the respective re-isolates was performed on the bases of comparisons of the obtained RAPD-PCR patterns for each individual strain and its respective re-isolates before and after the artificial infection of the plants. The type strain *C. flaccumfaciens* pv. *oortii* CFBP 1384 was included as the control. The extracted cDNA from all initial isolates and re-isolates were amplified with primer CUGEA 6 (5′–GGA AGC TTC G–3′) [45]. The reactions were conducted in a total final volume of 25 µL, containing 16.5 µL of sterile ultra-pure water, 6.5 µL of VWR Red Taq polymerase master Mix (VWR International bvba/sprl, Haasrode Researchpark Zone 3, Geldenaaksebaan 464 B-3001, Haasrode Belgium), 1 µL of primer CUGEA 6 and 1 µL of the DNA sample. The PCR reactions were performed in the MJ Research PTC-200, Peltier Thermal Cycler, under the following conditions: initial denaturation at 95 °C for 10 min, followed by 30 cycles of denaturation at 94 °C for 1 min, 42 °C for 1 min and 72 °C for 1.3 min. The final elongation was at 72 °C for 10 min. The resulting amplicons were separated and visualized by 2% agarose gel electrophoresis for 50 min at 100 V.

## 3. Results

### 3.1. Plant Samples and Isolation of the Bacteria

**Figure 2:**
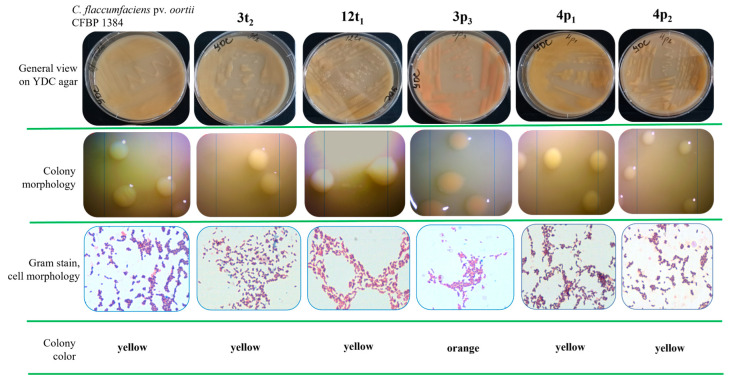
Morphological characterization of the isolates.

Recently, during a routine study aimed at the isolation, characterization and identification of phytopathogenic bacteria associated with bacterial spot and canker on solanaceous vegetables (*C. michiganensis* subsp. *michiganensis*, *X. euvesicatoria* pv. *euvesicatoria*, *X. euvesicatoria* pv. *perforans*, *X. vesicatoria* and *X. gardneri*) in Bulgaria, five other isolates (designated as 3t_2_, 12t_1_, 3p_3_, 4p_1_ and 4p_2_) with a similar macromorphology to the species mentioned above were also isolated. The isolates were obtained from different parts of tomato and pepper plants showing symptoms of bacterioses (Figure 1). Strain 3t_2_ was isolated from sample T3 (tomato stem), strain 12t_1_ was isolated from sample T12 (tomato seeds originating from sample T8, representing ripe mature tomato fruit collected from the same vegetable field), strain 3p_3_ was isolated from sample P3 (sweet pepper leaf) and strains 4p_1_ and 4p_2_ were isolated from sample P4 (chili pepper leaf) (Figure 1). Our preliminary analyses showed that these isolates did not belong to any of the common pathogens mentioned above. The morphology of the colonies on the YDC agar of all isolates were very close to those of type strain *C. flaccumfaciens* pv. *oortii* CFBP 1384. Four of the isolates (3t_2_, 12t_1_, 4p_1_ and 4p_2_) formed yellow-pigmented and one (3p_3_) formed orange-pigmented smooth and shiny colonies (Figure 2). 

### 3.2. Phenotypic Characterization of the Isolates

All strains were Gram-positive (Figure 2), with a coryneform cell morphology (short rods), and were catalase-positive and oxidase-negative. The strains were strictly aerobic, capable of hydrolyzing esculin, starch and casein, and negative for urease and indole production. None of the tested strains had the ability to reduce nitrates and nitrites. The obtained results showed that the isolates can be attributed to the group of phytopathogenic coryneform bacteria [20,22].

### 3.3. Identification of the Isolates

All five isolates were identified as *C. flaccumfaciens* by MALDI-ToF MS. The 16S rDNAs obtained from our strains were sequenced and processed to clear the low-quality regions, and after that were deposited at the GenBank with approved accession numbers (AN) (Table 1). The bioinformatic analyses of our 16S rDNA sequences showed that the five strains shared very high similarity (>99%) with the species *C. flaccumfaciens* pv. *flaccumfaciens* strain 1037, GenBank accession number CP041259.1 [46] (according to the NCBI), and with *C. flaccumfaciens* type strain LMG 3645, GenBank accession number AJ312209 (according to EzBioCloud) [7]. The score values (SVs) from the MALDI-ToF and the similarity percentages obtained after nucleotide blast analyses are shown in Table 1. 

**Figure 3:**
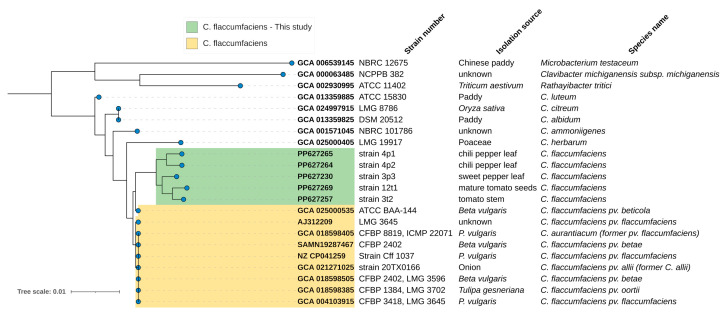
Phylogenetic tree constructed based on 16S rDNA gene alignment, comparing *C. flaccumfaciens* strains isolated by us and reported in this study to other species within the genus *Curtobacterium* and family *Microbacteriaceae*.

Additionally, a phylogenetic analysis based on 16S rDNA gene alignment, comparing our *C. flaccumfaciens* strains reported in this study to other members the genus *Curtobacterium* and family *Microbacteriaceae*, was made. The results showed that our strains clustered together with *C. flaccumfaciens* and had less distance to the *C. flaccumfaciens* species compared to other *Curtobacterium* species. This corresponded with the morphological examinations and MALDI-ToF (Figure 3).

Plasmid DNAs were successfully isolated from all of our isolates as well as from the type culture *Cfo*. For the PCR amplification with primers CffFor2/CffRev4, chromosomal and plasmid DNAs were used as templates separately. The obtained results showed that positive amplifications (approximately 306 bp of amplified product) were observed in the plasmid DNAs of all tested strains except for strain 12t_1_. No amplification products were observed in the agarose gel electrophoresis when cDNA was used as template in the PCR reactions.

### 3.4. Evaluation of the Ability of Newly Isolated C. flaccumfaciens Strains to Produce AMSs

**Figure 4:**
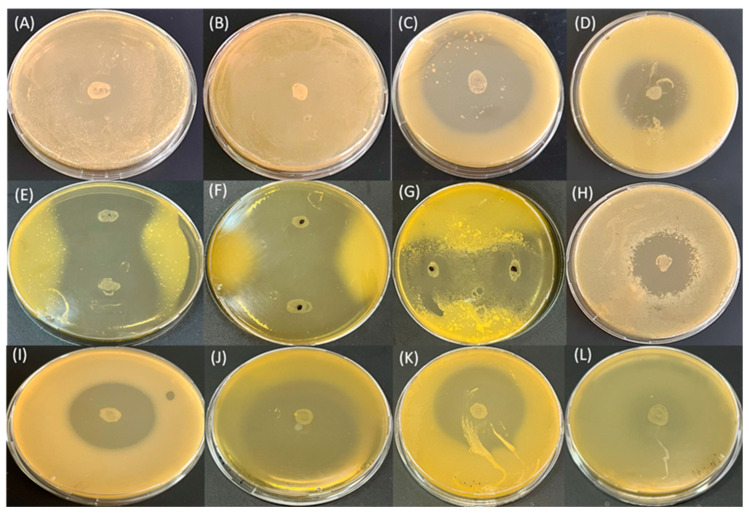
Production of antimicrobial substances (AMSs) from *C. flaccumfaciens* isolates against various test bacteria. Spot cultures in the center of the Petri dishes are AMS producers. (**A**) *Cf* 3p_3_ (AMS producer), *Cmm* CFBP 2492 (test microorganism); (**B**) *Cf* 4p_1_ (AMS producer), *Cmm* CFBP 2492 (test microorganisms); (**C**) *Cf* 3p_3_ (AMS producer), *X. gardneri* 62t (test microorganism); (**D**) *Cf* 12t_1_ (AMS producer), *X. euvesicatoria* 105t (test microorganism); (**E**) *Cf* 4p_1_ (AMS producer), *X. vesicatoria* 124t (test microorganism); (**F**) *Cf* 4p_2_ (AMS producer), *X. euvesicatoria* 105t (test microorganism); (**G**) *Cf* 3t_2_ (AMS producer), *X. vesicatoria* 124t (test microorganism); (**H**) *Cf* 3p_3_ (AMS producer), *P. putida* ATCC 12633 (test microorganism); (**I**) *Cfo* CFBP 1384 (AMS producer), *P. putida* ATCC 12633 (test microorganism); (**J**) *Cfo* CFBP 1384 (AMS producer), *X. euvesicatoria* 269p (test microorganism); (**K**) *Cfo* CFBP 1384 (AMS producer), *X. vesicatoria* 124t (test microorganism); (**L**) *Cfo* CFBP 1384 (AMS producer), *Cf* 12t_1_ (test microorganism).

The newly isolated *C. flaccumfaciens* strains and the type strain *C. flaccumfaciens* pv. *oortii* CFBP 1384 were tested for the ability to produce AMSs against Gram-negative and Gram-positive bacteria. The obtained results showed that all isolates inhibited the growth of all tested phytopathogenic and plant-associated bacteria to varying degrees (Figure 4). The susceptibility of Gram-negative bacteria to the AMSs produced by the investigated *C. flaccumfaciens* strains was greater than that observed for the Gram-positive test bacteria. 

All tested Gram-negative bacteria showed SS or IS to the AMSs produced by our *Cf* strains and by *Cfo* CFBP 1384. The largest inhibition zones (70 mm) were formed by *Cf* strains 3t_2_ and 12t_1_ (isolated from tomato), and by *Cfo* CFBP 1384 against the phytopathogenic bacteria *X. gardneri* strain 62t and *X. euveicatoria* strain 269p (Table 2). These two species of Gram-negative phytopathogenic bacteria were found to be the most sensitive to AMSs produced by the producer strains. The ability of *Cf* strains isolated from pepper (3p_3_, 4p_1_ and 4p_2_) to inhibit the growth of the tested the phytopathogenic and plant-associated bacteria was also very strong, although the inhibition zones were smaller than those formed by *Cf* strains isolated from tomato (3t_2_ and 12t_1_). The plant-associated species *P. putida* ATCC 12633 showed IS to AMSs produced by all *Cf* strains (Figure 4). The species *C. michiganensis* subsp. *michiganensis* CFBP 2492 (*Cmm*) showed resistance to almost all AMSs produced by the *Cf* isolates, as only one isolate (3p_3_) and *Cfo* CFBP 1384 inhibited its growth (inhibition zones of 20 and 35 mm, respectively).

The evaluation of the antibacterial activity of the *Cf* isolates against each other showed that the strain 4p_2_ had the most pronounced effect against four of the isolates—12t_1_, 3p_3,_ 4p_1_ and 3t_2_. The first three of them had intermediate sensitivity and the fourth (3t_2_) had weak sensitivity to the AMSs produced by strain 4p_2_ (Table 2). In turn, isolate 4p_2_ was resistant to the action of the AMSs synthesized by three isolates (3t_2_, 3p_3_ and 4p_1_) and weakly sensitive to the action of strain 12t_1_. In general, the majority of the *Cf* strains showed a low sensitivity to each other and to the AMSs synthesized by them. We also found that the isolates 4p_1_ and 4p_2_ differed from each other in the antibacterial activity of the AMSs produced by them, as well as in their susceptibility to the AMSs produced by the other producing isolate, despite having a common origin (chili pepper leaf). 

### 3.5. Determination of RAPD-PCR Profiles of the C. flaccumfaciens Isolates

**Figure 5:**
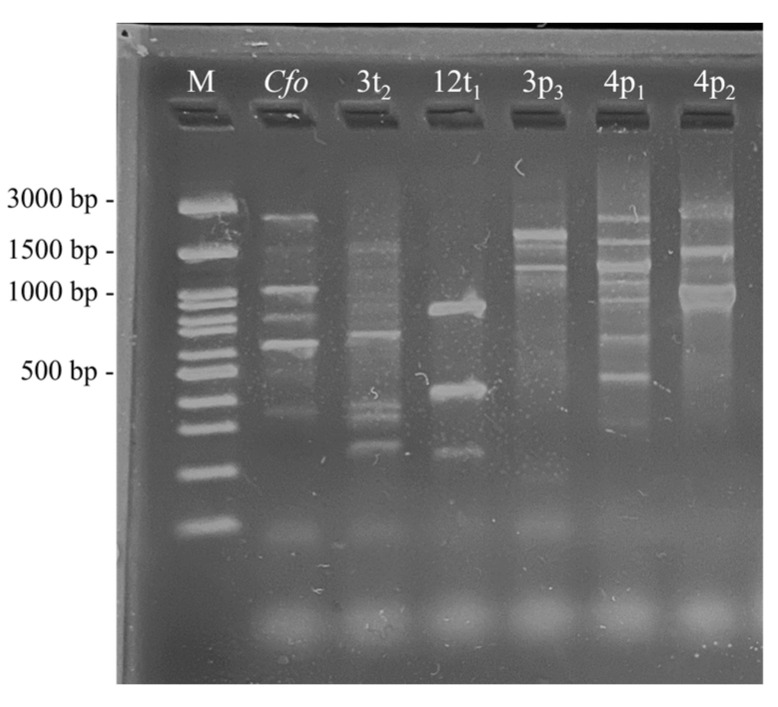
RAPD–PCR patterns obtained after the amplification of the cDNA of all *Cf* isolates and *C. flaccumfaciens* pv. *oortii* CFBP 1384 (*Cfo*) with primer CUGEA 6. M—molecular weight marker (SERVA FastLoad 100 bp DNA Ladder, Heidelberg, Germany).

RAPD-PCR analysis was performed in order to establish the specific amplification profiles of our *C. flaccumfaciens* isolates. This molecular approach was optimized for the purposes of our study for an easy, simple, reliable and fast method for the routine detection of the isolates in artificially infected plants. In the preliminary experiments carried out by us, the RAPD-PCR assays were performed with four CUGEA primers (CUGEA 3, 4, 5 and 6) [45] in order to determine the most suitable primer for strain discrimination. CUGEA 6 was chosen as the most discriminative and was used in the subsequent analyses. The resulting RADP profiles showed high pattern diversity (Figure 5). The amplification profiles of our *Cf* isolates differed significantly both from each other and from the profile of the type strain *Cf*o CFBP 1384. 

All tested strains formed different RAPD profiles, which means that this approach can be used as a reliable approach in future analyses aimed at the quick recognition of our strains among the surrounding microbiome of the plants after artificial infection and the re-isolation of the strains from the infected plants’ parts. The RAPD profile of the type culture *Cfo* was described by six fragments (each with an approximate length (bp) of 350, 600, 800, 1000, 1500 and 2800). The profiles of our *Cf* isolates, expressed in bp, were as follows: *Cf* 3t_2_—four fragments (250, 300, 350 and 600), *Cf* 12t_1_—three fragments (200, 400 and 800), *Cf* 3p_3_—five fragments (500, 1300, 1500, 1600 and about 3000), *Cf* 4p_1_—seven fragments (400, 600, 800, 900, 1000, 1500 and 2800) and *Cf* 4p_2_—three fragments (1000, 1400 and 2800).

### 3.6. Artificial Infection of Solanaceous Plants and Re-Isolation of the Inoculated Cf Strains

**Figure 6:**
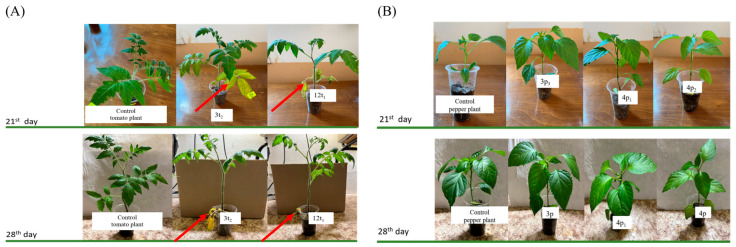
(**A**) Artificially infected tomato plants and (**B**) pepper plants. Red arrows indicate the yellowing of the first two to four leaves on the tomato plants and the defoliation at the 28th day.

Tomato and pepper plants were artificially infected with the strains of the putative pathogen *Cf* we isolated to determine their ability to survive, multiply and persist in the vascular system of these alternate host plants. It was found that, after the 7th day from the beginning of the experiment, the following morphological changes were observed in the tomato plants infected with strains 3t_2_ and 12t_1_: yellowing of the first two leaves and the appearance of yellow spots on them, as well as the appearance of wilting. These symptoms increased over the next two weeks. At the 28th day, defoliation of the first four leaves and the first two leaves in the tomato plants infected with the strains 3t_2_ and 12t_1_, respectively, was observed (Figure 6). Such symptoms were not observed in the artificially infected pepper plants, and by the end of the examined period, no worrisome morphological changes were observed.

After the cultivation period (28 days), all plants were analyzed to detect and confirm the presence of the inoculated *Cf* strains in them. All aboveground parts of the plants (leaves, stems and petioles) were examined separately. After the incubation of the prepared plant samples on the YDC agar, colonies corresponding to those of the original strains (yellow- or orange-pigmented, smooth and shiny) were observed only in the stem samples obtained from the tomato and pepper plants. Little variation in colony morphology was found on those Petri dishes, most of which were comparable to the colonies of the initial isolates. A total of 73 cultures were re-isolated from single colonies: 16 from stem samples from plants infected with strains 12t_1_, 3p_3_, 4p_1_ and 4p_2_, separately, and 9 from stem samples of tomato plants infected with strain 3t_2_. Re-isolates were designated as follows: 3t_2_/1 to 3t_2_/9; 12t_1_/1 to 12t_1_/16; 3p_3_/1 to 3p_3_/16; 4p_1_/1 to 4p_1_/16; 4p_2_/1 to 4p_2_/16.

### 3.7. Determination of the Similarity between Re-Isolates and the Initial Cf Strains

**Figure 7:**
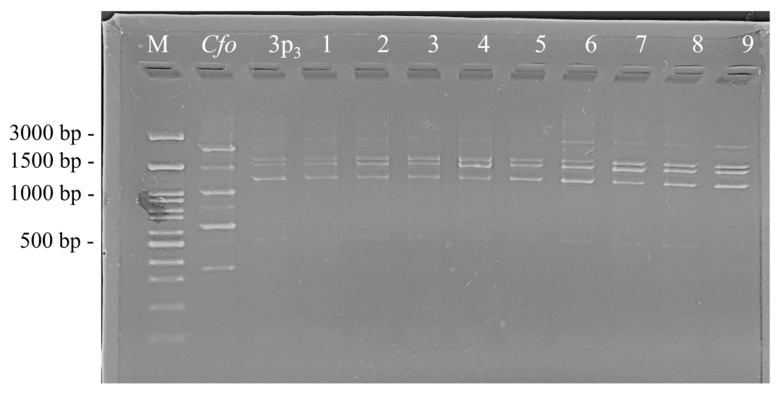
RAPD-PCR patterns of selected re-isolates. 1 to 9—respective re-isolates (3p_3_/1 to 3p_3_/9); *Cfo—*the type strain *C. flaccumfaciens* pv. *oortii* CFBP 1384; 3p_3_—the original *Cf* strain; M—molecular weight marker (SERVA FastLoad 100 bp DNA Ladder, Heidelberg, Germany).

The observation under light microscope showed that all re-isolates were Gram-positive. The morphology of the cells was the same as that of the corresponding original *Cf* strains, which is typical of the coryneform bacteria. The re-isolates were strictly aerobic, catalase-positive and oxidase-negative. The similarity between all 73 re-isolates and the corresponding original strains used for the artificial infection of the test plants was established at the molecular level based on the comparison between the obtained RAPD profiles after the PCR amplification with the primer CUGEA 6. The results showed complete identity between the RAPD profiles of the respective re-isolates and those of the original *Cf* strains. In Figure 7, the RAPD profiles of the selected re-isolates (3p_3_/1 to 3p_3_/9) obtained from the stem samples of pepper plants infected with strain *Cf* 3p_3_ and compared to the RAPD profile of the initial *Cf* 3p_3_ strain are shown.

## 4. Discussion

The ability of plant pathogenic bacteria to persist, survive and retain pathogenic potential in habitats different from their primary hosts is their important feature enabling them to expand their host range. The role of the alternative and alternate host plants as natural reservoirs of phytopathogenic bacteria has recently been widely discussed. The term “alternative” refers to the hosts that are usually members of the same family as the primary hosts of a particular pathogen, while “alternate” is used when the host is from different family [47]. Many of them reside on various plants without causing disease symptoms. These are the cases with *X. perforans* and *X. gardneri* isolated from weeds [48], *X. vesicatoria* isolated from symptomless weeds associated with tomato production areas [49] and pathogenic *Curtobacterium* strains isolated from alfalfa, maize, sunflower, tomato, pepper, eggplant and wheat [22,24]. Such residents can rearrange their pathogenicity mechanisms and induce disease symptoms on new susceptible plants, as was the case with two *C. flaccumfaciens* strains causing chlorosis and leaf death on maize under greenhouse experiments [22]. Since maize is not one of *C. flaccumfaciens*’ primary hosts, this shift in the bacterial pathogenicity is quite concerning and has prompted the need for additional research in this area.

The present investigation was conducted with plant samples (showing symptoms of microbial diseases) collected in our previous study in a private vegetable garden, where various vegetables and fruits were grown [41]. Five strains (3t_2_, 12t_1_, 3p_3_, 4p_1_ and 4p_2_) not belonging to the common casual agents of bacterial spot and canker diseases were isolated. The observed morphological characteristics and established phenotypic features corresponded with those reported for representatives of the species *C. flaccumfaciens* and its several pathovars [20,22,50]. Except the yellow- and orange-pigmented colonies, the species was reported to also produce red or pink colonies, and also variants of yellow colonies producing a diffusible water-soluble purple pigment [24,50,51].

The phenotypic characterization of our five isolates revealed that they possessed some of the typical metabolite features reported for the coryneform plant pathogens. Usually, the biochemical features of the strains are not solid bases for their distinguishing, either at the pathovar level or the species level. However, some characteristics (casein hydrolyses) have been used for additional differentiation inside the species *C. flaccumfaciens*. It has been reported that two pathovars of the species are positive for this feature: the yellow-pigmented strains of pv. *flaccumfaciens* and pv. *oortii* [14]. Interestingly, the newly described species *C. aurantiacum* (formerly *C. flaccumfaciens* pv. *flaccumfaciens*), forming pink-, red- and orange-pigmented colonies, has been reported to be negative for this characteristic, as well as *C. poinsettiae* (formerly *C. flaccumfaciens* pv. *poinsettiae*) [14,52]. However, in other studies, red-pigmented variants of the species *C. flaccumfaciens* (now *C. aurantiacum*) have been reported to be positive for this phenotypic feature [22]. Among our strains, there is also one orange colony variant, strain *Cf* 3p_3_, which is positive for casein hydrolysis. Thus, according to the new taxonomy inside the species *C. flaccumfaciens*, this strain could be considered as close to either the species *C. aurantiacum* or *C. poinsettiae* based on this phenotypic feature [14].

The species identification of our strains was performed by a combination of three approaches. According the results obtained from the MALDI-ToF MS, all strains were identified as *C. flaccumfaciens*. Over the years, the MALDI–ToF MS has been used as a reliable approach for the identification of *C. flaccumfaciens* and *Cff* in particular [17,28,29]. These results were confirmed by sequencing analyses of the 16S rDNA of our strains, revealing an above 99% similarity with the *C. flaccumfaciens* pv. *flaccumfaciens* strain 1037 and *C. flaccumfaciens* LMG 3645, respectively [7,46]. The same percent identity was detected with one more species, *C. allii*, in the EzBioCloud database. This is not surprising, as the latter species was recently reclassified as a pathovar of the species *C. flaccumfaciens* [14]. The use of 16S rDNA as an approach in the identification of *Curtobacterium* strains has been also reported before [25,27,28,29,30]. Our phylogenetic analyses showed that our strains clustered closer to *C. flaccumfaciens* than to any other representatives of the genus *Curtobacterium*.

The issue for the presence and the role of plasmids in *C. flaccumfaciens* pathogenicity has been discussed before [53]. For example, the pathogenicity of *C. flaccumfaciens* pv. *flaccumfaciens* strain 1037 on *P. vulgaris* has been demonstrated, and the role of a linear plasmid pCFF113, carrying genes related to the pathogenicity, has been proposed [46]. We investigated the presence of plasmids in the genome of our strains and established their presence in all strains as well as in the type culture of *Cfo*. Earlier studies, have reported the absence of plasmids in the genomes of a large number of strains of *C. flaccumfacien* pv. *flaccumfaciens* when studied via classical agarose gel electrophoreses [15]. Recent studies based on the sequencing of the genome of one of these strains (P990) have revealed that it contains three plasmids, pCff1, pCff2 and pCff3 [53]. Furthermore, it has been proposed that one of these plasmids (pCff1) harbors genes related to the pathogenicity of the strain, including serine protease, pectate lyase and others [53].

The third method used for the identification of our strains was PCR with the primers CffFor2/CffRev4, targeting a conserved region of the genome of the *Cff* strains [31]. Our results showed that the expected amplification product (approximately 306 bp) was visualized in the agarose gel electrophoresis only when the plasmid DNA was used as a template in the PCR reactions, and not when cDNA was used. The only exception was strain 12t_1_, where no amplification products were observed in both the plasmid and cDNA samples. Based on our results, we could suggest that these primers are designed on plasmid DNA.

Our preliminary bioinformatic research in the NCBI database showed that the primers mentioned above and used in this study hybridize specifically to a particular region in the plasmids, which were detected in the genomes of five *Cff* strains (the accession numbers of the plasmids are as follows: CP041260.1 (plasmid pCff113), CP045288.2 (plasmid pCff1), CP080396.1 (unnamed plasmid), CP074440.1 (plasmid pCff119) and CP071884.1 (plasmid Cff119)). Thus, it could be proposed that positive amplification is expected only if the strain contains plasmids harboring this particular region. These findings are in accordance with our results, suggesting that our strains carry the plasmid of interest. Moreover, no specific data concerning the role of the targeted sequence of these primers have been published. Only one suggestion for the similarity of the putative protein encoded by this sequence with serine proteases found in other species has been assumed [22]. Our bioinformatic research in the NCBI database revealed that the translated amino acid sequence provided a similarity to the trypsin-like serine protease or S1 family peptidase found in other *Curtobacterium* species.

Based on the results obtained from the three independent approaches, we can conclude that our strains belong to the species *C. flaccumfaciens*, with great possibility to be attributed to *C. flaccumfaciens* pv. *flaccumfaciens* (except strain 12t_1_). However, further studies on the pathogenicity of our strains on susceptible hosts should be carried out in order to establish their exact pathovar belonging.

To our knowledge, our study is the first report on the isolation of *C. flaccumfaciens* in Bulgaria at all and the first report of *C. flaccumfaciens* (strain 12t_1_) isolated from tomato seeds in particular. Furthermore, the newly isolated strains were obtained in tomato and pepper plants, which are not among the known susceptible hosts of this bacterium. We found no reported data on the isolation of *Cf* from tomato seeds. The majority of the papers reported the epiphytic or endophytic persistence of *Cf* in non-host plants and especially on solanaceous vegetables [22,54]. Previous studies aimed at establishing the accompanying microflora (pathogenic, epiphytic and endophytic) of tomato flowers with symptoms of bacterioses have also been conducted before in Bulgaria, but no data on the isolation of *C. flaccumfaciens* have been reported [55]. In our research, we found that *Cf* strains coexisted with various species in plant tissues. In the tomato stem sample (T3), the strain 3t_2_ was found to coexist with *Pseudomonas* and *X. euvesicatoria* (data not published). In the seed sample (T12), the strain 12t_1_ was detected along with *P. agglomerans* and *Enterobacter cloacae* [41]. In the pepper leaf sample (P3), the isolate 3p_3_ coexisted with *P. agglomerans*, *R. larrymoorei* and *X. euvesicatoria* [41]. The chili pepper leaf sample (T4) was found to be inhabited only by *Cf* strains 4p_1_ and 4p_2_, as reported in this study.

An important feature of actinobacteria is the production of various antimicrobial substances [56]. The representatives of genus *Curtobacterium* as actinobacterial species have been also reported to produce antimicrobial substances [22,57]. The antibacterial action of two *Curtobacterium* strains against representatives of the so-called “ESKAPE” group *(Enterococcus faecium*, *Staphylococcus aureus*, *Klebsiella pneumoniae*, *Acinetobacter baumannii*, *Pseudomonas aeruginosa* and *Enterobacter species*) have been demonstrated [56]. The deep investigation of such substances derived from ten *Cff* strains revealed that they belong to a group of temperature-resistant compounds, bacteriocins, and have been named flaccumfacin. Additionally, it has been proposed that the bacteriocins produced from the bacterial wilt pathogen may contribute to its pathogenicity [57]. According to more recent research, the *Cff* strains’ aggressiveness on common beans positively correlated with their possibility to produce bacteriocins [52]. Thus, the ability of our isolate to produce AMSs, as established in this study, could be considered a key characteristic defining their putative pathogenicity on susceptible and alternate hosts.

In order to enable the implementation of more effective management strategies, in-depth studies on the role of alternative and alternate host plants as natural reservoirs in the lifecycle of phytopathogenic bacteria should be carried out. In this regard, the artificial infection of tomato and pepper plants in this study aimed to establish the ability of our *Cf* isolates to live and multiply in the vascular systems of plants, which were so far not reported as susceptible host of this bacterium. We also aimed to clarify whether they have accidentally come upon these plants or can inhabit them and therefore use them as natural reservoirs. By inoculating our *Cf* strains into the xylem of the plants, we aimed to determine whether any morphological changes would develop in the aerial parts of the plants and whether the strains would be viable and successfully recovered after the examined period. A similar study investigating the role of various weeds as natural reservoirs of *Cff* was conducted by Nascimento et al. [58]. The authors reported that the *Cff* strain Feji 2628A can survive in the phyllosphere and rhizosphere of several weed species for up to 70 days [58]. Another similar study reported that solanaceous vegetables could also serve as sources for the isolation of *Cf* strains, i.e., the bacteria persist on them without affecting the plants [22]. In that study, the pathogenic capacity of *Cff* strains was investigated on several crop species, including tomato, bell pepper, eggplant, maize, common bean, mung bean, cowpeas, soybean, zucchini, faba bean, honeydew melon, rapeseed, sugar beet, sunflower, etc. [22]. However, there was no evidence of the successful re-isolation of *Cff* strains from artificially infected tomatoes, peppers and eggplants. Expectedly, in that study, *Cff* strains were successfully re-isolated from the primary host plants as well as from symptomless plants [22].

Unlike in our study, during the investigated period (28 days), we observed the appearance of morphological changes only in tomato plants infected with strains 3t_2_ and 12t_1_, but not in the pepper plants. Our assumption was that the persistence of *Cf* in the vascular system of tomato plants may have an additional impact on the plant’s physiological condition. Furthermore, we successfully re-isolated viable *Cf* strains from the stem parts of the test plants 28 days after their artificial infection. Our results showed that the 73 re-isolates shared phenotypic features and molecular characteristics, including RAPD-PCR patterns with the original *Cf* strains that were used for the artificial infection the test plants. RAPD–PCR is a molecular tool that is considered to be highly accurate in identifying a strain’s genome uniqueness, which is useful when a certain strain’s behavior needs be tracked in a variety of contexts. The unique molecular structure of a strain’s genome, identified by RAPD amplification, provides an accurate, fast and straightforward method for monitoring its presence in plants after artificial infection, all without the risk of mistaking the strain with one that might exist in the plant naturally.

## 5. Conclusions

Numerous questions arise regarding the pathways of plant pathogens and the capacity to adapt their pathogenicity mechanisms to inhabit new ecological niches and expand their host range. The effective management of plant diseases requires in-depth knowledge of the natural reservoirs of plant pathogens. Thus, studies investigating the ability of phytopathogenic bacteria to exploit alternate host plants as reservoirs for survival and spread to susceptible host plants is very important.

To our knowledge, our study is the first report on the isolation and characterization of *C. flaccumfaciens* in Bulgaria, as well as the first report of its isolation from tomato seeds. The characterization of our isolates showed that they possessed the biological features which refer them to the group of phytopathogenic coryneform bacteria. We demonstrated that tomato and pepper plants can act as reservoirs of this pathogen, finding that it successfully survives in these plants. The morphological changes we observed in the artificially infected tomato plants were close to the symptoms of bacteriosis and raises the question of the pathogenicity of these strains to this plant. More in-depth studies are needed in order to clarify the molecular response of tomato plants to infection with *C. flaccumfaciens*. We believe that the results obtained from the present study could be a good basis for further in-depth research in this area.

## Figures and Tables

**Table 1:** Species identification of the isolated strains.

Isolates	GenBank AN	MALDI-TOF		16S rDNA Sequencing, Percent Identity	
		Identification	SV	Closest *Cf* Strain [7]	Closest *Cff* Strain [46]
3t_2_	PP627257	*C. flaccumfaciens*	1.97	99.4%	99.2%
12t_1_	PP627269		2.10	99.3%	99.4%
3p_3_	PP627230		2.04	99.7%	99.5%
4p_1_	PP627265		2.14	99.0%	99.2%
4p_2_	PP627264		2.08	99.6%	99.2%

**Table 2:** Action of antimicrobial substances (AMSs) produced by *C. flaccumfaciens* isolates, reported in this study, against Gram-negative and Gram-positive bacteria.

Test Microorganisms	AMS Producers, Inhibition Zones (mm)					
	*Cf* 3t_2_	*Cf* 12t_1_	*Cf* 3p_3_	*Cf* 4p_1_	*Cf* 4p_2_	*Cfo* CFBP 1384
Gram-negative bacteria						
*X. euvesicatoria* 40f	57	60	30	50	58	60
*X. euvesicatoria* 269p	39	70	45	60	60	60
*X. euvesicatoria* 105t	65	40	50	30	50	50
*X. vesicatoria* 124t	38	40	46	45	50	50
*X. gardneri* 62t	70	70	58	43	48	70
*P. putida* ATCC 12633	35	33/MS	35	30	30	37
Gram-positive bacteria						
*Cmm* CFBP 2492	0	0	20	0	0	35
*Cf* 3t_2_	NA	19	12	15	20	20
*Cf* 12t_1_	18	NA	11	10	33	20
*Cf* 3p_3_	14	18	NA	18	23	20
*Cf* 4p_1_	13	14	11	NA	21	10
*Cf* 4p_2_	4	16	6	4	NA	6
*Cfo* CFBP 1384	25	25	11	17	20	NA

Purple color, ≥41 mm, strong sensitivity; pink color, 21–40 mm, intermediate sensitivity; yellow color, 7–20 mm, weak sensitivity; green color, 0–6 mm, resistance. NA—not applicable.

## Data Availability

The data presented in this research are available in the manuscript.
